# Diet-Independent Correlations between Bacteria and Dysfunction of Gut, Adipose Tissue, and Liver: A Comprehensive Microbiota Analysis in Feces and Mucosa of the Ileum and Colon in Obese Mice with NAFLD

**DOI:** 10.3390/ijms20010001

**Published:** 2018-12-20

**Authors:** Eveline Gart, Everton Souto Lima, Frank Schuren, Christa G. F. de Ruiter, Joline Attema, Lars Verschuren, Jaap Keijer, Kanita Salic, Martine C. Morrison, Robert Kleemann

**Affiliations:** 1 Department of Metabolic Health Research, The Netherlands Organization for Applied Scientific Research (TNO), 2333 CK Leiden, The Netherlands; christa.deruiter@tno.nl (C.G.F.d.R.); joline.attema@tno.nl (J.A.); kanita.salic@tno.nl (K.S.); martine.morrison@tno.nl (M.C.M.); Robert.kleemann@tno.nl (R.K.); 2 Human and Animal Physiology, Wageningen University, 6708 WD Wageningen, The Netherlands; jaap.keijer@wur.nl; 3 Department of Microbiology and Systems Biology, The Netherlands Organization for Applied Scientific Research (TNO), 3704 HE Zeist, The Netherlands; everton.soutolima@tno.nl (E.S.L.); frank.schuren@tno.nl (F.S.); lars.verschuren@tno.nl (L.V.); 4 Department of Vascular Surgery, Leiden University Medical Center, 2333 ZA Leiden, The Netherlands

**Keywords:** non-alcoholic fatty liver disease, obesity, liver, short-chain fatty acids, gut permeability, adipose tissue inflammation

## Abstract

Development of non-alcoholic fatty liver disease (NAFLD) is linked to obesity, adipose tissue inflammation, and gut dysfunction, all of which depend on diet. So far, studies have mainly focused on diet-related fecal microbiota changes, but other compartments may be more informative on host health. We present a first systematic analysis of microbiota changes in the ileum and colon using multiple diets and investigating both fecal and mucosal samples. Ldlr−/−.Leiden mice received one of three different energy-dense (ED)-diets (*n* = 15/group) for 15 weeks. All of the ED diets induced obesity and metabolic risk factors, altered short-chain fatty acids (SCFA), and increased gut permeability and NAFLD to various extents. ED diets reduced the diversity of high-abundant bacteria and increased the diversity of low-abundant bacteria in all of the gut compartments. The ED groups showed highly variable, partially overlapping microbiota compositions that differed significantly from chow. Correlation analyses demonstrated that (1) specific groups of bacteria correlate with metabolic risk factors, organ dysfunction, and NAFLD endpoints, (2) colon mucosa had greater predictive value than other compartments, (3) correlating bacteria differed per compartment, and (4) some bacteria correlated with plasma SCFA levels. In conclusion, this comprehensive microbiota analysis demonstrates correlations between the microbiota and dysfunctions of gut, adipose tissue, and liver, independent of a specific disease-inducing diet.

## 1. Introduction

Non-alcoholic fatty liver disease (NAFLD) has become a major health problem worldwide [1]. Ample evidence shows that an increased intake of calories from saturated fat and carbohydrates such as sucrose and fructose contribute to the development of NAFLD [2,3]. Furthermore, important drivers of the disease are dysmetabolism (e.g., dyslipidemia, hyperinsulinemia), white adipose tissue (WAT) inflammation [4], and gut dysfunction [5,6]. Gut dysfunction includes dysbiosis, alterations of microbiota-derived metabolites such as short-chain fatty acids (SCFAs), and increased gut permeability [7]. Together, these dysfunctions are thought to propel the progression of NAFLD from the relative benign hepatic lipid accumulation (steatosis) toward non-alcoholic steatohepatitis (NASH), which is the severe stage of NAFLD that is characterized by steatosis and liver inflammation [8].

The reported alterations of the gut microbiota in obese and NAFLD/NASH patients are typically based on the analysis of the fecal colonic microbiota, which is an easily accessible gut site. However, it is known that different bacteria dominate in the various regions of the intestinal tract [9,10], and that the microbiota of the fecal and mucosal compartment is composed of different bacteria [11]. Hence, it is possible that an analysis of specific gut microbial compartments may be more informative on host health than the conventional analysis, which is solely based on fecal microbiota. However, a systematic analysis of multiple microbial compartments of the gut in relation to obesity and NAFLD endpoints has not been performed to date.

In the present study, we examined microbiota changes evoked by energy-dense (ED) diets in the colon and ileum during obesity-associated NAFLD development. In the colon and ileum, fecal and mucosal compartments were analyzed by 16S rRNA gene sequencing. We complemented this with the analysis of SCFAs, which constitute potential mediators between the microbiota and host health. We studied NAFLD development in Ldlr−/−.Leiden mice through an established translational model for NAFLD/NASH [12,13,14]. Upon the feeding of ED diets, these mice developed metabolic dysfunction and features of human NAFLD (obesity, dyslipidemia, insulin resistance, WAT inflammation, hepatic steatosis, lobular inflammation, and fibrosis) [12].

Since diets can alter microbiota composition [15], we evaluated the generic effects of three different ED diets containing macronutrients that are frequently present in human diets, i.e., diets rich in saturated fat (butter or lard), as well as rapidly metabolizable carbohydrates (fructose and sucrose). These three diets were compared with a reference chow-fed group, which did not develop obesity and NAFLD. The chosen approach allowed us to investigate general diet-independent changes in specific gut microbial compartments that inform on readouts of metabolic dysfunction and NAFLD development (e.g., fat mass, WAT inflammation, gut permeability, macrovesicular steatosis, hepatic inflammation). We demonstrate that specific bacteria correlate with these readouts, independent of the NAFLD-inducing diet used, and that these correlations differ between gut compartments and involve different bacteria in different compartments.

## 2. Results

### 2.1. All ED Diets Resulted in NAFLD in the Context of Obesity, White Adipose Tissue Dysfunction, and Gut Dysfunction

**Figure 1:**
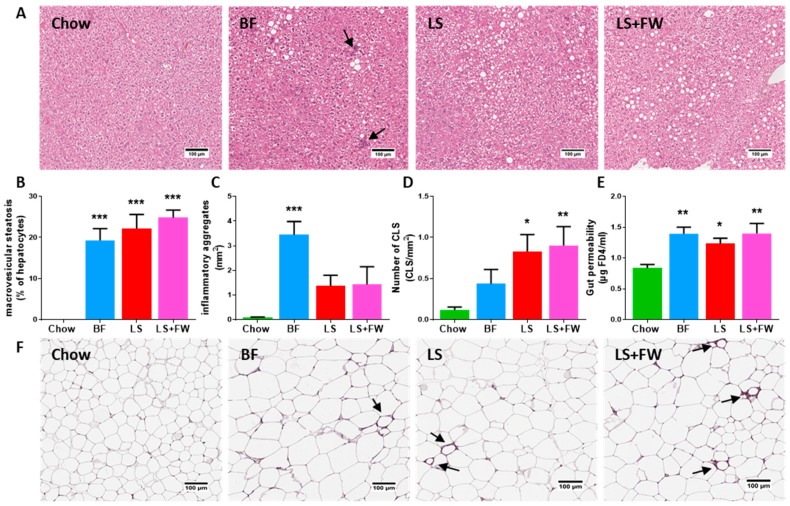
Different energy-dense diets induced liver and white adipose tissue (WAT) dysfunction, and was associated with increased gut permeability. (**A**) Representative photomicrographs of HE-stained liver sections of chow, butter fat–fructose (BF), lard fat–sucrose (LS), and a diet with LS and fructose water (LS+FW); arrows indicate inflammatory aggregates. Histological quantification of (**B**) macrovesicular steatosis and (**C**) inflammatory aggregates per mm^2^ in the liver and (**D**) crown-like structures (CLS) per mm^2^ in WAT; (**E**) Functional gut permeability analysis using a fluorescein isothiocyanate-labeled dextran (FD4) assay; (**F**) Representative photomicrographs of hematoxylin-phloxine-saffron-stained gonadal WAT sections, arrows indicate CLS. Data are presented as mean ± SEM, * *p* ≤ 0.05 or ** *p* ≤ 0.01, or *** *p* ≤ 0.001 compared to the chow control group.

After 15 weeks, all of the ED diets induced hepatic steatosis, as shown in Figure 1A. More specifically, quantitative histopathological analysis revealed that a butter fat with fructose diet (BF), lard fat with sucrose diet (LS), and the same LS diet with 10% fructose in the drinking water (LS+FW) induced extensive macrovesicular steatosis (Figure 1B) relative to chow-fed controls (0.0 ± 0.0% in chow; 19.3 ± 2.8% in BF, *p* < 0.001; 22.1 ± 3.5%, *p* < 0.001 in LS; 24.8 ± 1.9% in LS+FW, *p* < 0.001). Hepatic inflammation, which was expressed as number of inflammatory aggregates per mm^2^ (Figure 1C), was induced by all of the ED diets in varying degrees, which reached statistical significance in BF-fed mice (3.4 ± 0.5 per mm^2^, *p* < 0.001). Furthermore, plasma levels of the liver damage marker alanine aminotransferase (ALT) were significantly increased in BF; while in LS, a slight (non-significant) increase was observed, and levels were significantly increased in LS+FW (Table 1).

These characteristics of liver dysfunction were observed in the context of an obese phenotype with pronounced hyperinsulinemia, hypertriglyceridemia, and hypercholesterolemia (Table 1). Body weight was significantly increased relative to the chow-fed controls after 15 weeks on all of the ED diets. Blood glucose on the ED diets did not significantly differ from the chow-fed controls; however, insulin was markedly elevated, with all the ED reaching statistical significance in the LS and LS+FW diets indicating a reduction in insulin sensitivity (Table 1). Furthermore, plasma cholesterol and triglycerides (TG) levels were higher in the ED diet groups, and BF was the strongest inducer of dyslipidemia (Table 1).

In line with the observed increase in body weight, total fat mass was significantly increased on the ED diets (Table 1) compared with the control diet. In addition to this increase in adipose tissue mass, quantitative histological analysis of inflammation in the gonadal white adipose tissue depot (representative images shown in Figure 1F) demonstrated that the number of CLS in the BF group was comparable to chow (Figure 1D) (0.1 ± 0.0 in chow; 0.4 ± 0.2 in BF, *p* = 0.45). By contrast, treatment with LS and LS+FW resulted in pronounced CLS formation in WAT (0.8 ± 0.2, *p* = 0.02; 0.9 ± 0.2, *p* = 0.03).

In addition, gut barrier function measured with an fluorescein isothiocyanate-labeled dextran (FD4) assay for gut permeability (Figure 1E) was significantly elevated on BF, LS, and LS+FW relative to chow (0.8 ± 0.1 in chow; 1.4 ± 0.1 in BF, *p* < 0.001; 1.2 ± 0.1, *p* = 0.04 in LS; 1.4 ± 0.2 in LS+FW, *p* < 0.001). Furthermore, the plasma levels of gut-derived metabolites (SCFAs) were quantified by high-performance LC-MS (Table 2). These metabolites may be potential bioactive mediators in host health, and 90% are absorbed from the gut [16]; therefore, their plasma levels may be more informative than conventionally studied fecal levels. Plasma levels of the SCFA propionate were markedly elevated in LS-fed mice only, while acetate and butyrate were reduced in BF and LS+FW. Plasma levels of the SCFAs isobutyrate, methylbutyrate, isovalerate, and valerate (also known as the isoacid SCFAs) were all significantly increased in LS and LS+FW.

Collectively, these data show that all of the ED diets induced NAFLD in varying degrees of severity in the context of obesity, WAT inflammation, and gut dysfunction.

### 2.2. Global Analysis of Fecal and Mucosal Microbiota Composition in Colon and Ileum

To investigate whether the observed diet-induced metabolic dysfunction in liver, white adipose tissue, and gut may be linked to changes in bacterial colonization in specific gut compartments, we performed a 16S rRNA gene microbiota analysis of the fecal and mucosal compartments of both the colon and ileum.

**Figure 2:**
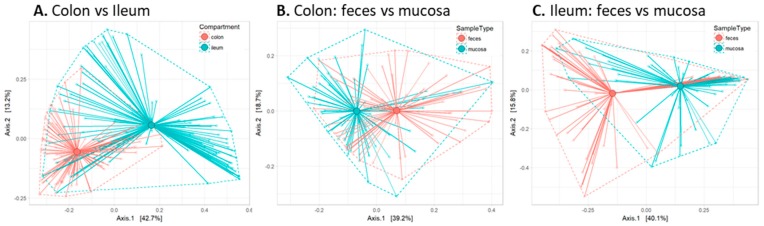
Refined microbiota analysis reveals marked composition differences between the gut compartments. Permutation tests were used to calculate statistical differences in microbiota composition, and group distances were visualized between (**A**) the colon and ileum; (**B**) colon feces and mucosa; and (**C**) ileum feces and mucosa.

First, we investigated whether these four gut compartments differ in their colonization using permutation tests, which enable the statistical testing of differences between the groups. This analysis revealed a clear difference in microbiota composition between the colon and ileum (Figure 2A, *p* < 0.05), which is consistent with their different physiological roles. Furthermore, we observed significant differences between the mucosal and the fecal compartments in both regions of the gut (Figure 2B, colon *p* < 0.05; Figure 2C, ileum *p* < 0.05).

**Figure 3:**
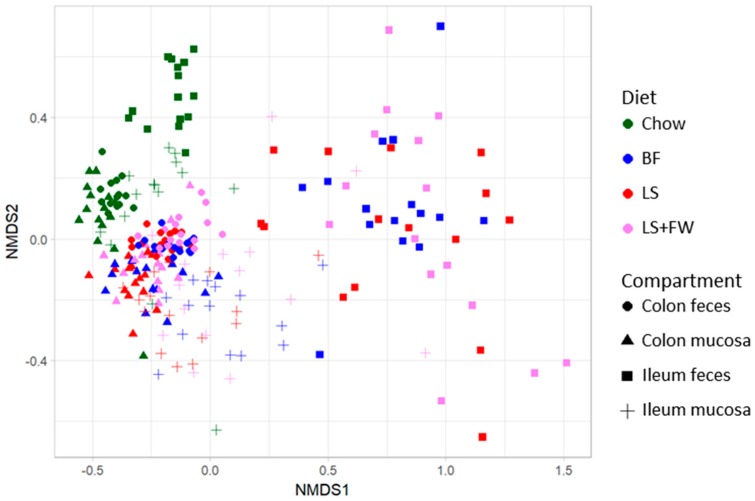
Effects of the non-alcoholic fatty liver disease (NAFLD)-inducing energy-dense diets on microbiota composition of the colon and ileum in both the feces and mucosa. *16S rRNA* gene sequencing analysis was performed on the luminal and mucosal contents of both the ileum and colon, and was used to visualize microbiota ordination with non-metric multidimensional scaling (NMDS) using the Bray–Curtis index. Complementary statistical analysis were performed with permutation tests and visualized in the previous figure to show the differences in microbiota composition between gut compartments. The following figure shows the differences in microbiota composition between the energy-dense diets.

**Figure 4:**
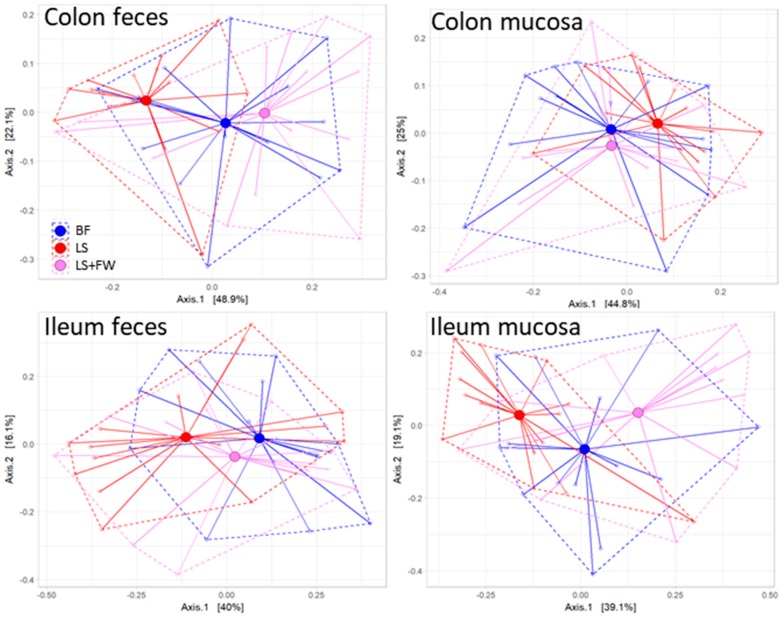
Microbiota composition between the energy-dense diets was comparable in all of the gut compartments. Permutation tests were used to calculate statistical differences in microbiota composition, and group distances were visualized between ED diets in the colon feces, colon mucosa, ileum feces, and ileum mucosa.

Next, we investigated whether the microbiota composition was affected by ED feeding and whether there were differences in microbiota composition between the three ED diet groups, in which the dissimilarity in composition was visualized with non-metric multidimensional scaling analysis (NMDS). This NMDS plot (Figure 3) demonstrated that the chow animals formed a discrete cluster, in which ileum feces differed the most from the other compartments. The microbiota composition of the ED diet groups was clearly different from that of the chow diet group (i.e., did not overlap with the chow cluster), and showed pronounced variation with no clear separation of the different ED diets. Similarly to what was observed in chow-fed animals, the ileum feces compartment was the most dissimilar and most variable in comparison with the other compartments. These observations were statistically confirmed by permutation tests, which revealed that the microbiota composition of the chow group clearly differed from the other groups in all of the compartments (*p* = 0.002) (see also Figure S1), whereas the composition of the ED diets was statistically comparable in all of the compartments (Figure 4; colon feces *p* = 0.09, colon mucosa *p* = 0.32, ileum feces *p* = 0.60, ileum mucosa *p* = 0.07).

**Figure 5:**
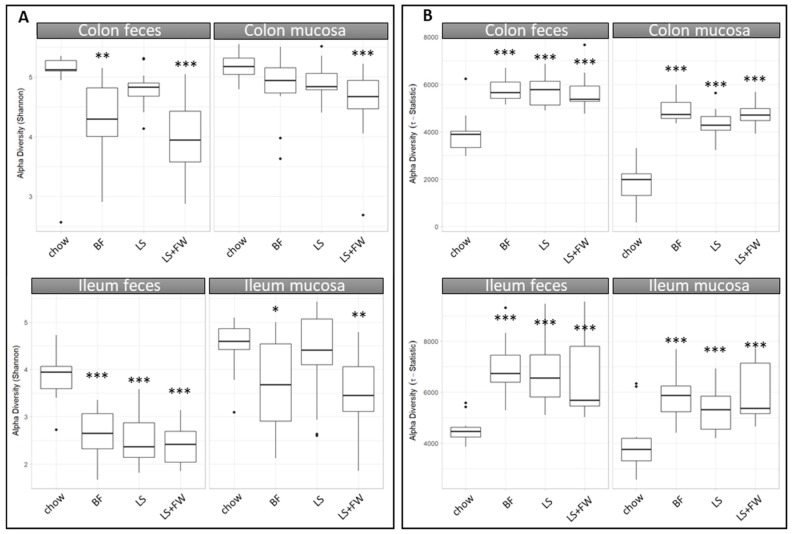
All of the energy-dense (ED) diets reduced diversity in high-abundant bacteria and increased diversity in low-abundant bacteria in all of the gut compartments. 16S rRNA gene sequencing analysis was performed on the luminal and mucosal contents of both the ileum and colon, and was used to calculate alpha diversity using the (**A**) Shannon index (sensitive for high-abundant bacteria) and (**B**) the tail statistic (sensitive for low-abundant bacteria). Data are presented as mean ± SD, * *p* ≤ 0.05, ** *p* ≤ 0.01, or *** *p* ≤ 0.001 compared to chow.

To gain more insight into how the microbiota of the ED diets differed from those of the chow-fed controls, we next investigated the effects of the NAFLD-inducing diets on microbiota diversity using the Shannon index (sensitive to high-abundant bacteria) and tail-statistic analysis (sensitive to low-abundant bacteria). The Shannon index analysis demonstrated that the ED diets consistently reduced the microbiota diversity of high-abundant bacteria in all of the compartments that were studied (Figure 5A), which reached statistical significance by BF in colon feces, ileum feces, and ileum mucosa (*p* = 0.01; *p* < 0.001; and *p* = 0.02), LS only in the ileum feces (*p* < 0.001), and LS+FW in all of the compartments (*p* < 0.001; *p* < 0.001; *p* < 0.001; and *p* < 0.001). This reduction in diversity was more pronounced in the feces than in the mucosa in both the colon and ileum, suggesting a more robust microbiota in the mucosal compartments compared with feces. Complementary to this analysis, the Pielou evenness index was calculated, which represents species evenness (Figure S2). A high number indicates that an equal number of bacteria belong to each species, while a low number indicates the presence of dominant species. The results of the Pielou evenness index show a similar trend as the Shannon index, and confirm that the ED diets reduce species diversity. In contrast, the tail-statistic analysis showed that the diversity of low-abundant bacteria was increased by the ED diets in all of the compartments (Figure 5B) (*p* < 0.001 for all of the ED diets in all of the gut compartments). These results suggest that abundant bacteria decreased, while low abundant bacteria increased on the ED diets.

**Figure 6:**
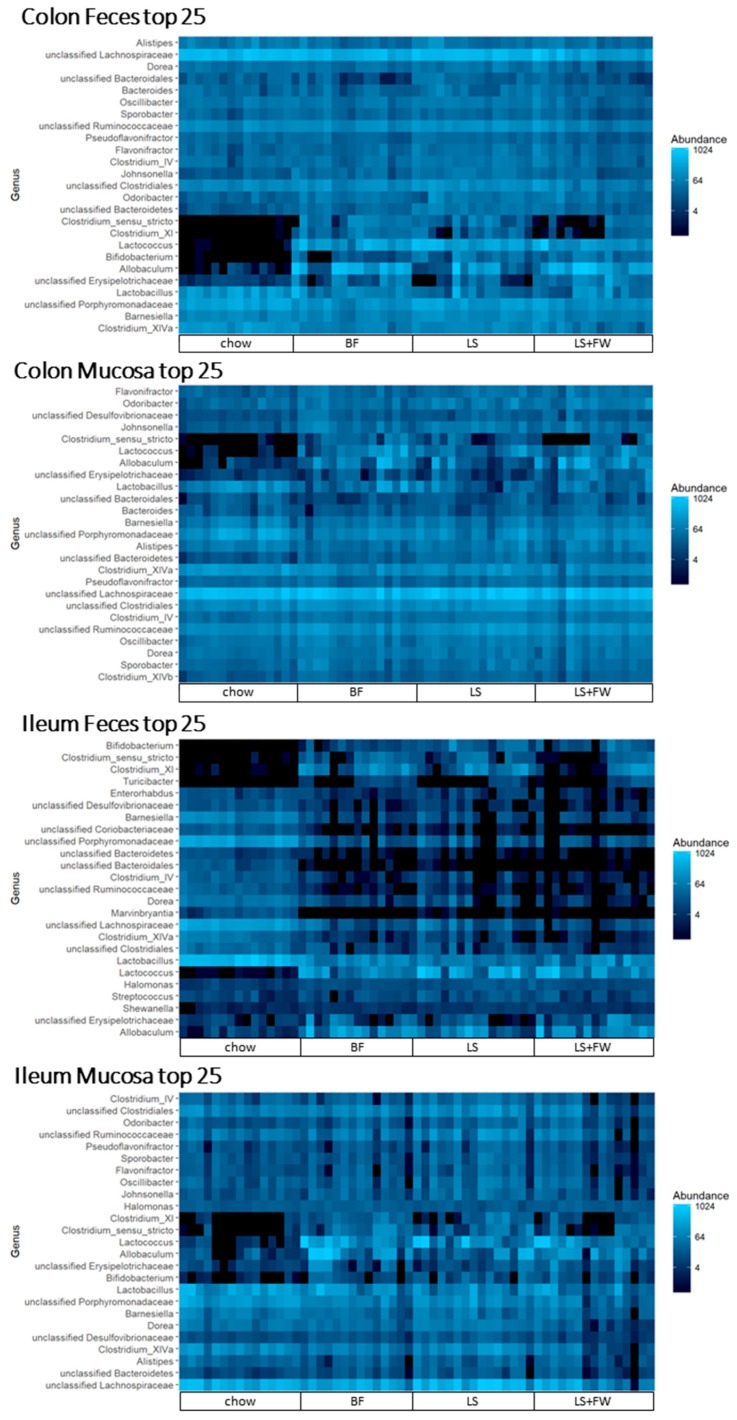
Diet-independent changes of the microbiota composition after 15 weeks of energy-dense diet treatment. 16s rRNA gene sequencing analysis was performed on the luminal and mucosal contents of both the ileum and colon, and the abundance of the 25 most dominant genera were expressed in a heat map.

In order to study general disease-associated microbiota changes, independent of the specific ED diet used, we investigated the microbiota abundance on the genus level in all four gut compartments using heat maps as a visualization tool (Figure 6). These heatmaps illustrate that several of the top most abundant genera increased in abundance on all of the ED diets, independent of the gut microbial compartment, including *Lactococcus*, *Allobaculum*, *Bifidobacterium*, *Clostridium sensu stricto*, and *Clostridium_XI*.

Altogether, these analyses show that there were significant differences in composition between the gut microbial compartments studied, and that the three NAFLD-inducing ED diets resulted in a microbiota composition that is significantly different from chow, but did not differ between the ED diets, thus allowing us to study diet-independent disease-associated changes in the microbiota composition.

### 2.3. Microbiota Composition Associations with NAFLD Disease Endpoints

Next, we investigated whether there are correlations between the microbiota composition and histological and functional readouts of metabolic dysfunction and NAFLD development (body weight, fat mass, WAT inflammation, gut permeability, macrovesicular steatosis, hepatic inflammation) or plasma levels of gut-derived metabolites (SCFAs: acetate, propionate, butyrate, methylbutyrate, isobutyrate isovalerate, and valerate), independent of the specific ED diet that was employed. For this, we performed a comprehensive regularized canonical correlation analysis (rCCA), which allows the study of relationships between sets of variables. This analysis revealed that correlations exist between the gut microbiota composition and histological and functional readouts in all of the compartments that were studied (Table 3). The strength of this correlation (expressed in the cross-validation score (CV score), which is a total (weighted) correlation score, where a higher score implies a stronger relation) was highest for the colon mucosa (CV scores of 0.71 for endpoints and 0.62 for metabolites), while colon feces (the typically used sampling compartment) showed weaker correlations (CV scores of 0.56 and 0.43 for endpoints and metabolites, respectively). In contrast, in the fecal compartment of the ileum, both the endpoints and metabolites correlated better (CV scores of 0.60 and 0.62, respectively) than the mucosal compartment (CV scores of 0.52 and 0.41, respectively).

**Figure 7:**
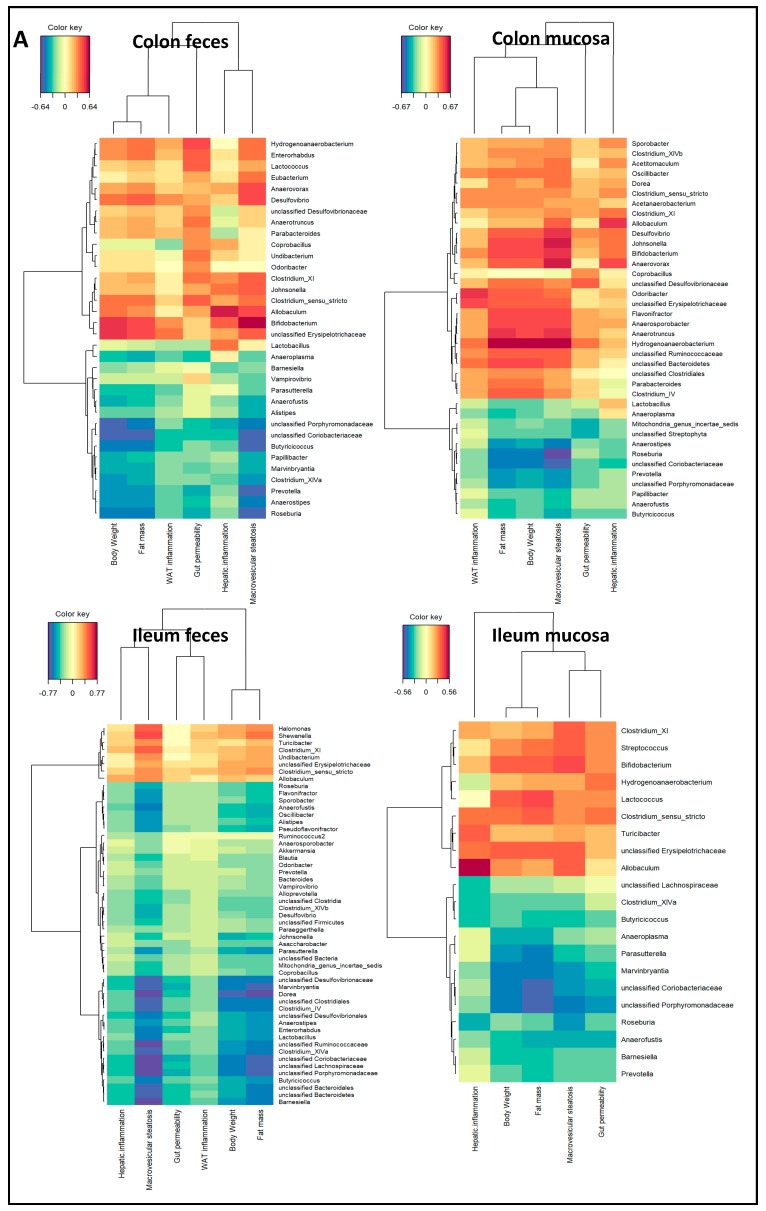

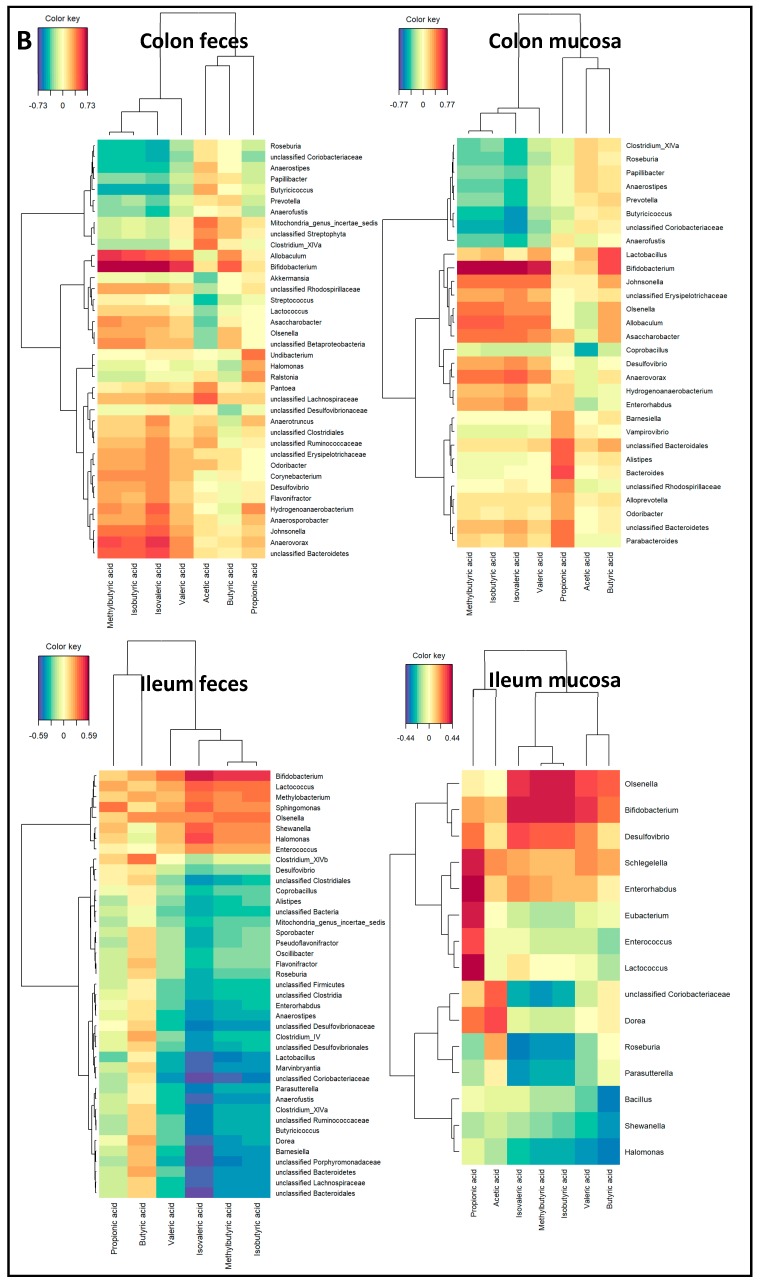
Diet-independent correlations between the microbiota and NAFLD-associated readouts or gut-derived metabolites. Regularized canonical correlation analysis (rCCA analysis) results were visualized in a heat map for positive (red) and negative (blue) correlations between the microbiota composition and (**A**) histological and functional readouts of non-alcoholic fatty liver disease (NAFLD) development or (**B**) gut-derived metabolites for each gut compartment analyzed. Correlations with CV scores >0.03 are shown.

Consistent with our previous finding that each compartment exhibited a different microbiota composition, bacteria with the strongest correlations differed, to a large extent, in each of the compartments. However, correlation analysis between microbiota and histological and functional readouts showed strong positive correlations for *Allobaculum* and *Bifidobacterium*, and strong negative correlations for the *Roseburia* and *Anaerostipes* bacteria, in all of the compartments (Figure 7A). Interestingly, correlation analysis between the microbiota and plasma levels of gut-derived metabolites (Figure 7B) revealed that the bacteria that positively correlated with disease readouts (indicating an unfavorable metabolic state) also positively correlated with the isoacid SCFAs (isobutyrate, methylbutyrate, isovalerate, and valerate).

Altogether, we identified diet-independent correlations between specific bacteria and the histological and functional readouts of NAFLD development, in which the strongest correlations were found for the colon mucosa compartment.

## 3. Discussion

The objective of the present study was to investigate whether there are generic changes in specific gut microbial compartments that correlate with metabolic risk factors, organ dysfunction, and NAFLD endpoints, independent of the specific energy-dense diet (ED diet) that was used to induce disease. We demonstrated that all of the ED diets induced obesity and NAFLD during 15 weeks of ED diet feeding, but the severity of liver pathology, WAT dysfunction, and gut dysfunction varied between the individual ED diets. Microbiota composition analysis revealed significant differences between the gut compartments, but not between the ED diets. The diversity of highly abundant bacteria decreased, while the diversity of low-abundant bacteria increased on the ED diets. Independent of the type of ED diet used, we identified correlations between certain bacteria and readouts of metabolic dysfunction and NAFLD development, and correlating bacteria differed per gut compartment. The colon mucosa had the greatest predictive value when compared with the other compartments. Interestingly, plasma levels of SCFAs (isoacids) may function as a possible reflective marker of disease-associated microbiota changes and a less beneficial metabolic state.

We found that all of the ED diets that were used induced NAFLD, but diets differed with respect to the severity of liver pathology that was induced. The pronounced induction of liver inflammation with BF cannot be explained by an increase in caloric intake or gut permeability in this group, because the other ED diets had a comparable caloric intake and developed a similar level of gut permeability. Thus, it is likely that hepatic inflammation in BF is a consequence of the quality of calories, i.e., a macronutrient-mediated effect. One possible explanation may be the difference in the type of carbohydrates in the ED diets; the BF diet only contains fructose, while LS and LS+FW are high in sucrose (i.e., consisting of both fructose and glucose). Furthermore, fructose intake was higher (1.3 g of fructose/day) in animals on the BF diet than in the other ED diet groups (0.5 g/day in LS and 0.9 g/day in LS+FW). Studies have shown that while both fructose and glucose are able to induce hepatic steatosis and steatohepatitis [17], liver injury is typically much worse in fructose-treated mice [17,18], which is in line with the findings herein. Another possible explanation for the pronounced hepatic inflammation observed with BF is the fat source of the diet: BF contains butter fat, while LS and LS+FW contain lard. Butter fat contains more saturated fat than lard, and saturated fatty acids (SFA) have been shown to induce liver inflammation [19,20].

The choice of the diet was made based on previous studies with these diets (unpublished data), and the knowledge that these diets would induce different levels of obesity, adipose tissue inflammation, and NAFLD pathology in this mouse model. Furthermore, many NASH studies use experimental diets that are difficult to translate to human diets, because experimental diets are supplemented with supraphysiological levels of cholesterol (e.g., 2% *w*/*w* cholesterol) or experimental diets with extremely high fat content are used (e.g., >50% energy from fat). The diets that were used in this study were not supplemented with dietary cholesterol, and contained a level of dietary fat that can also be found in traditional diets, for instance those of Finland and Greece ([21] and references therein). A recent study showed that the LS diet employed herein results in a plasma metabolome and a liver transcriptome disease profile that recapitulates the metabolome and gene expression profile of NASH patients [13,22], which supports the use of such diets, the more so because more extreme diets did not induce human-like gene expression changes [23].

The NAFLD-inducing ED diets also significantly altered the composition of the microbiota relative to chow. However, the composition within the ED diet groups was very variable, and there was no significant difference between the three ED diets in microbiota composition. These findings may seem unexpected, since dietary macronutrient composition is thought to be a very important determinant of microbiota composition [15,24]. However, earlier studies did not compare the total microbiota composition between diet treatments, but rather focused on specific bacterial changes by macronutrients [25,26,27,28]. Other reasons for the absence of significant differences between the ED diets used herein may be a) that not all of the macronutrients were changed (e.g., the protein source was casein in all of the ED diets) and b) that the relative content of each macronutrient (e.g., protein, carbohydrate, fat) was comparable among the ED diets. Consistent with this view, the macronutrient composition of the chow-control diet did markedly differ from the ED diets, and chow-fed mice also had a microbiota that significantly differed from the ED diets.

The diversity of the microbiota was also similarly affected by the three ED diets, with a reduction in the diversity of high abundant bacteria in all of the gut compartments. These findings are consistent with previous findings using high-fat diets in both humans [29,30] and mice [30,31]. Yet, while these studies only focus on the fecal compartment, we now also show that this reduction in diversity also occurs in the mucosal compartments of the ileum and colon. Conversely, we observed an increase in the diversity of low-abundant bacteria. This is in line with studies that focused on microbiota abundance during aging [32,33], which is accompanied by a general decline in health status and metabolic disease development. These studies, together with our observations, indicate that the reduction in diversity of high-abundant bacteria is a generic pattern in all gut compartments during metabolic disease development. Together with an increase in the diversity of low-abundant bacteria, this may serve as an indicator of an unfavorable metabolic state. In addition, we observed that the mucosal microbiota composition was more stable in terms of diversity than the fecal compartment, which may be a consequence of the functional differences between gut compartments [34,35]. The mucosal microbiota is in closer proximity to the gut barrier; therefore, it is assumed that these bacteria (and their metabolites) are in more direct contact with the host than the fecal microbiota [36].

A strength of this study is that we used three different NAFLD-inducing ED diets, allowing us to study correlations between the microbiota and NAFLD that are not dependent on one specific disease-inducing diet. We found that there are correlations between the microbiota and readouts of metabolic dysfunction and NAFLD development, which were strongest for the colon mucosa. Remarkably, we observed a strong positive correlation of *Bifidobacterium* with NAFLD-associated readouts, suggesting an adverse role. The observed positive correlation between *Bifidobacterium* and NAFLD endpoints may be related to specific strains of *Bifidobacterium*, as shown by Yin et al. [37] in a rat model of HFD obesity. The authors found that body weight development upon *Bifidobacterium* treatment is strain-dependent, suggesting that different strains may affect energy metabolism and fat distribution in a different way. Furthermore, an open label pilot trial in adult human subjects with NAFLD (*n* = four) showed that participants treated with the probiotic VSL#3 (which also contains *Bifidobacterium*) experienced a significant increase in liver fat after four months, and this was reversed in three subjects after washout [38]. The authors concluded that the results of this trial did not support their hypothesis (probiotics would reduce hepatic steatosis in humans). Notably, the effect of HFD on *Bifidobacterium* abundance also varies among studies, because some studies report an increased abundance upon HFD feeding [25], while others report a reduction in its abundance [30,39]. Similarly, the role of *Lactobacillus* is unclear: Jiang et al. showed that the abundance of *Lactobacillus* was increased in the microbiota of NAFLD patients [40], suggesting a role in disease development, whereas probiotic treatment with *Lactobacillus* has been associated with beneficial effects in clinical NAFLD trials [6]. Hence, associative findings should be treated with caution, and even correlations should be interpreted carefully. The positive correlations between bacteria and endpoints on the genera level that were observed in our study indicate a tight relationship between gut microbiota and NAFLD development, yet follow-up intervention studies with specific species (e.g., *Bifidobacterium* strains as in Yin et al. [37]) are necessary to define the disease-augmenting or disease-attenuating role of particular bacteria to demonstrate their causal role. It is possible that the composition of bacterial species that underlie a correlation on genera level may change in response to an intervention (and remain unnoticed on genera level), but may result in an altered production of metabolites and altered gut functioning.

We found that increased gut permeability positively correlated with higher levels of the *Hydrogenoanaerobacterium* in the colon feces and mucosa. In this study, we measured gut permeability after four hours of fasting, which is a time span that mainly reflects colonic permeability based on time-resolved analyses with the tracer (FD4) used herein [41]. Associations between gut permeability and *Hydrogenoanaerobacterium* have not been reported before. However, *Hydrogenoanaerobacterium* is increased in fecal microbiota in both obese mice [42] and humans [43], which is consistent with our findings.

Interestingly, our correlation analyses revealed that bacteria that were positively correlated with disease-associated readouts were also strongly positively correlated with the isoacid SCFAs (isobutyrate, methylbutyrate, isovalerate, and valerate) of which physiological effects have hardly been investigated so far. Isoacids are a product of protein fermentation by the microbiota, for which branched-chain amino acids (positively associated with insulin resistance and metabolic disease development [44,45]) are one of the sources to synthesize them [46]. Hence, the elevated plasma isoacids levels that were observed herein may indicate enhanced protein fermentation, which is associated with the production of potential toxic by-products, and has been linked to intestinal disease [47,48].

## 4. Materials and Methods

### 4.1. Animals, Diets, and Study Design

Ethics approval: Approval was granted by the ethics committee on animal experiments (approval reference number DEC-3682, 11 December 2014) and the institutional animal welfare body (approval reference number TNO-136, 29 March 2016).

All of the animal experiments were performed in accordance with the Animal Care and Use Committee of The Netherlands Organization of Applied Research (TNO), Leiden, The Netherlands. All of the mice were group-housed (four to five mice per cage) in Macrolon cages in clean-conventional animal rooms in the American Association for Accreditation of Laboratory Animal Care (AAALAC)-accredited animal facility at TNO Leiden (relative humidity 50–60%, temperature ~21 °C, light cycle 07:00 to 19:00) and had *ad libitum* access to food and water. Male Ldlr−/−.Leiden mice were obtained from the breeding facility of TNO Metabolic Health Research, Leiden, The Netherlands, and were kept on a low-fat control diet (chow; Sniff-R/M-V1530, Uden, The Netherlands, containing 23 kcal% protein (soy and cereal grains), 67 kcal% carbohydrate (mainly starch), 9 kcal% fat (grain cereals and soy products) and 4.9 *w*/*w*% fiber) until the start of the study (six weeks of age). The mice were then matched into four groups (*n* = 15/group) based on body weight and blood glucose. The first group remained on chow, while the other groups were switched to energy-dense (ED) diet regimens, as shown in Table 4. For a total of 15 weeks, groups of mice were fed a butter fat with fructose diet (BF; D16032401, Research Diets Inc., New Brunswick, NJ, USA; with 14 kcal% protein, 44 kcal% carbohydrate (mainly fructose), and 41 kcal% butter fat, 5.1 *w*/*w*% fiber) or a lard fat with sucrose diet (LS; D12451, Research Diets Inc.; with 20 kcal% protein, 35 kcal% carbohydrate (mainly sucrose), 45 kcal% lard fat, 5.8 *w*/*w*% fiber), or the same LS diet with 10% fructose in the drinking water (LS+FW). Body weight was measured at set intervals, and body composition was determined using echoMRI. After 12 weeks, gut permeability was analyzed using an FD4 test. In week 15, five-hour fasted tail blood plasma (Ethylenediaminetetraacetic acid; EDTA) was collected to measure cholesterol, TG, glucose, insulin, ALT, and SCFAs. After 15 weeks, five hour-fasted animals were terminated by gradual fill CO_2_ asphyxiation, and a terminal blood sample (for EDTA plasma) was collected by cardiac puncture. Isolated liver and epidydimal adipose tissue were fixed in formalin and paraffin-embedded for histological analysis. At sacrifice, microbiota from four different regions of the intestinal tract were sampled, i.e., the feces were collected and the mucosa was scraped in both the colon and ileum.

### 4.2. Blood Chemistry

Plasma lipids (cholesterol and TGs), ALT levels, and insulin were assessed in a fasted (five-hour) blood sample (100 µL, tail incision). Plasma total cholesterol and TGs were measured spectrophotometrically with enzymatic assays (Roche diagnostics, Almere, The Netherlands) according to the manufacturer’s instructions. Plasma ALT levels were measured by reflectance photometry (Reflotron-Plus system, Roche diagnostics). Blood glucose was measured during blood sampling using a hand-held glucose analyzer (Freestyle Disectronic, Vianen, The Netherlands). Plasma insulin levels were determined by ELISA (Mercodia, Uppsala, Sweden).

Plasma SCFAs were analyzed using ultra performance liquid chromatography (UPLC, Ultimate 3000 UPLC system, Thermo Scientific, Waltham, MA, USA) coupled to high-resolution mass spectrometry (HR-MS, Q-Exactive mass spectrometer equipped with an electro-spray ionization probe (Thermo Scientific)). Briefly, plasma was mixed with MilliQ water and deuterated internal standard solution was added. Subsequently, three M of zinc sulfate solution was used to precipitate proteins. After vortexing and centrifugation, the supernatant was used for derivatization with glycidyltrimethylammonium chloride solution (diluted in 100 mM of Tris-HCl buffer pH = 7.2) which was incubated for 90 min at 70 °C. After derivatization, the SCFAs were separated on an Acquity HSS T3 column (150 × 2.1 mm, 1.8 µm; Waters Corporation, Milford, MA, USA) using a mobile phase gradient from 99.5% mobile phase A (0.1% formic acid in MilliQ) to 100% mobile phase B (0.1% formic acid in acetonitrile). Mass detection was carried out using electrospray ionization in the positive mode (spray voltage three kV, scan range *m*/*z* 150–700).

### 4.3. Histological Analysis of Adipose Tissue and Liver

Paraffin-embedded cross sections (five-µm thick) of gonadal WAT were stained with hematoxylin-phloxine-saffron to blindly score the number of crown-like structures (CLS) in three non-overlapping fields (100× magnification) per mouse and data were expressed as number of CLS/mm^2^ [4]. Histopathological analysis of NAFLD was performed on five-µm thick hematoxylin-eosin stained cross-sections of the medial lobe. NAFLD was scored blindly by a board-certified pathologist using an adapted grading method for human NASH [12,49]. Briefly, two cross-sections per mouse were examined to assess the level of macrovesicular steatosis relative to the hepatocyte area, which was expressed as a percentage. Hepatic inflammation was evaluated by counting the number of inflammatory cell aggregates per field at a 100× magnification [12,50] in five non-overlapping fields per mouse, and expressed as the average number of cell aggregates per mm^2^.

### 4.4. FD4 Gut Permeability Assay

In vivo gut permeability was assessed by measuring the ability of the relatively impermeant fluorescein isothiocyanate-labelled dextran (three to five kDa FD4; Sigma, St. Louis, MO, USA) to cross from the intestinal lumen into the circulation, which is a test employed in human [51] and rodent studies [52,53]. Mice were fasted for four hours, after which a baseline blood sample was taken by tail incision. Then, FD4 was administered by oral gavage (900 mg/kg) and four hours later, a second blood sample was taken to determine the plasma concentration of FD4 using a fluorometer (FLUOstar Galaxy, BMG labtech, Offenburg, Germany). The baseline blood sample was used to correct for autofluorescence.

### 4.5. Statistical Analysis

Data is presented as mean ± standard error of the mean (SEM). Significance of differences between the control diet (chow) and the ED diet groups were tested using a one-way ANOVA with Dunnett’s post hoc test. Differences with *p* ≤ 0.05 were considered to be statistically significant. The statistics of the microbiota data analysis is described below.

### 4.6. Gut Microbiota Analysis

#### 4.6.1. Collection and DNA Isolation

Microbiota samples from both the colon and ileum collected from the feces or mucosa were collected at the end of the study and frozen immediately at −80 °C. Samples were mechanically homogenized and genomic DNA was isolated using the AGOWA mag mini kit (DNA Isolation Kit, AGOWA, Berlin, Germany) according to the manufacturer’s instructions.

#### 4.6.2. Metagenomic Sequencing and Data Analysis

A fragment of the 16S rRNA gene (~270 bp), spanning the V4 hypervariable regions, was amplified by PCR within 30 cycles using F515/R806 primers. Seventeen purified PCR products were paired-end sequenced on an Illumina MiSeq platform (Illumina, Eindhoven, The Netherlands). The sequences were classified by the RDP-II Naïve Bayesian Classifier (‘Wang method’) [54]. In addition, taxa that were not observed in at least 5% of the samples or had a total count of less than five were removed. Microbiota data analysis and visual representation was performed with R [55]: To address differences in sequencing depth and heteroscedasticity, the data was normalized by DESeq^2^ using the variance stabilization transformation [56]. Community profiles were analyzed on the genus level, compared by Bray–Curtis dissimilarity and visualized in non-metric multidimensional scaling (NMDS) plots. The minimum-entropy decomposition algorithm, which clusters 16S rRNA gene amplicons in a sensitive manner, was used to calculate the Shannon index, Pielou evenness index, and the tail-statistic for alpha diversity on the sequence level with the vegan package in R [57]. Significance of differences in diversity between chow and the ED diets was tested using a one-way ANOVA with Dunnett’s post hoc test. Statistical permutation tests were performed to compare composition level differences [58,59,60] with the phyloseq GraphTest package in R [61]. Correlation analysis between the microbiota composition and histological and functional readouts of NAFLD development were performed with regularized canonical correlation analysis (rCCA analysis) [62] using MixOmics in R [63].

## 5. Conclusions

Taken together, this first systematic analysis of different gut microbial compartments demonstrates with three different ED diets that a limited group of bacteria correlates with NAFLD endpoints. The comprehensive analysis of multiple gut compartments indicates that the colon mucosa may have added value above other compartments when it comes to prediction of the metabolic health state of organs such as the liver. Lastly, generic changes were observed in all of the compartments regarding microbiota diversity and changes in plasma SCFAs (isoacids), which may be used as a marker of the metabolic state.

## Figures and Tables

**Table 1:** Metabolic parameters. ALT: alanine aminotransferase.

	Chow	BF	LS	LS+FW
Body weight (g)	29.1 ± 2.2	34.5 ± 3.8 **	37.8 ± 6.0 ***	40.1 ± 5.1 ***
Total caloric intake (kcal/day)	11.0 ± 0.7	11.7 ± 1.1	11.5 ± 0.5	12.6 ± 0.8
Total fat mass (g)	3.7 ± 1.0	9.1 ± 1.9 ***	11.8 ± 4.0 ***	13.4 ± 4.8 ***
Plasma cholesterol (mM)	8.9 ± 1.8	50.9 ± 10.9 ***	20.3 ± 6.2 ***	24.3 ± 10.8 ***
Plasma triglycerides (mM)	1.6 ± 0.4	10.3 ± 2.4 ***	4.9 ± 1.9 ***	4.5 ± 2.6 ***
Plasma insulin (ng/mL)	1.3 ± 0.6	4.2 ± 1.2	4.6 ± 2.1 *	8.0 ± 6.8 *
Blood glucose (mM)	7.2 ± 1.0	6.3 ± 0.9	7.6 ± 2.1	7.7 ± 1.5 **
ALT (U/L)	32.6 ± 7.3	139.7 ± 76.4 ***	78.9 ± 50.0	137.1 ± 99.3 ***

Data are presented as mean ± SD, * *p* ≤ 0.05 or ** *p* ≤ 0.01 or *** *p* ≤ 0.001 compared to chow.

**Table 2:** Plasma levels of gut-derived metabolites (short-chain fatty acids, or SCFAs).

	Chow	BF	LS	LS+FW
Acetate (nM)	5966.6 ± 874.1	4828.2 ± 891.8 **	6468.3 ± 1361.3	4977.9 ± 810.9 **
Butyrate (nM)	123.7 ± 67.1	55.6 ± 14.4 **	142.3 ± 90.7	45.8 ± 7.3 **
Propionate (nM)	257.8 ± 103.3	310.8 ± 127.1	464.6 ± 214.9 **	388.3 ± 171.6
Isobutyrate (nM)	33.5 ± 7.3	45.7 ± 11.6	88.9 ± 42.7 ***	57.6 ± 12.2 *
Methylbutyrate (nM)	36.6 ± 8.5	46.9 ± 8.7	81.9 ± 34.6 ***	54.8 ± 8.9 *
Isovalerate (nM)	26.9 ± 4.6	33.3 ± 7.7	66.7 ± 16.7 ***	60.9 ± 19.8 ***
Valerate (nM)	10.8 ± 5.7	19.9 ± 11.8	36.4 ± 14.4 ***	23.7 ± 10.2 **

Data are presented as mean ± SD, * *p* ≤ 0.05, or ** *p* ≤ 0.01, or *** *p* ≤ 0.001 compared to chow.

**Table 3:** Correlation strength.

		Histological and Functional Readouts	Metabolites
Colon	Feces	0.56	0.43
	Mucosa	0.71	0.62
Ileum	Feces	0.60	0.62
	Mucosa	0.52	0.41

Data represents cross-validation score (CV score) from the regularized canonical correlation analysis (rCCA).

**Table 4:** Diet composition.

	Chow	BF	LS	LS+FW
Fat	9% kcal (Cereal grains and soy)	41% kcal (Butter fat)	45% kcal (Lard)	Same as LS
Protein	24% kcal (Soy and cereal grains)	14% kcal (Casein)	20% kcal (Casein)	Same as LS
Carbohydrate	67% kcal (Starch)	44% kcal (Fructose)	35% kcal (Sucrose)	Same as LS
Cholesterol	-	-	-	-
kcal/gram	3.2 kcal/g	4.5 kcal/g	4.7 kcal/g	Same as LS
Fiber	4.9% *w*/*w*	5.1% *w*/*w*	5.8% *w*/*w*	Same as LS
Drink	Water	Water	Water	10% *w*/*v* fructose in water

Ldlr−/−.Leiden mice were fed Chow, butter fat–fructose (BF), lard fat–sucrose (LS) or a diet with LS and fructose water (LS+FW) for 15 weeks (*n* = 15/diet grp). (-) = not present; (*w*/*w*) = weight per weight; (*w*/*v*) weight per volume; (% kcal) = percent of energy.

## Supplementary Materials

Supplementary materials can be found at http://www.mdpi.com/1422-0067/20/1/1/s1. Figure S1: Microbiota composition of chow differs from the ED diets. Figure S2: All of the ED diets reduced species evenness in all of the gut compartments. The 16S dataset can be accessed at https://dashin.eu/download/. For details, please contact the corresponding author.

## Abbreviations

NAFLD	Non-alcoholic fatty liver disease
NASH	Non-alcoholic steatohepatitis
Ldlr−/−	Low-density lipoprotein receptor knockout
ED	Energy-dense
BF	Butter fat fructose
LS	Lard fat sucrose
LS+FW	Lard fat sucrose and fructose water
HFD	High-fat diet
SCFA	Short-chain fatty acid
ALT	Alanine-aminotransferase
TG	Triglycerides
WAT	White adipose tissue
CLS	Crown-like structures
FD4	Fluorescein isothiocyanate-labeled dextran 4
LC-MS	Liquid chromatography–mass spectrometry
NMDS	Non-metric multidimensional scaling
CV-score	Cross-validation score
rCCA	Regularized canonical correlation analysis
IR	Insulin resistance
UPLC	Ultraperformance liquid chromatography
HR-MS	High-resolution mass spectrometry
