# Supplementary material for: Diet-Independent Correlations between Bacteria and Dysfunction of Gut, Adipose Tissue, and Liver: A Comprehensive Microbiota Analysis in Feces and Mucosa of the Ileum and Colon in Obese Mice with NAFLD
Source: Int J Mol Sci. 2018 Dec 20;20(1):1. doi: 10.3390/ijms20010001 (PMC6337295; doi:10.3390/ijms20010001)
Supplement: Supplementary file 1 [file ijms-20-00001-s001.pdf]

Supplementary materials

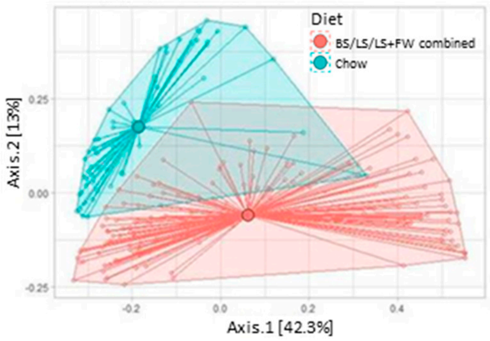

Figure S1. Microbiota composition of chow differs from the ED diets. Permutation tests were used to calculate statistical differences in microbiome composition and group distances were visualized between chow and all of the ED diets.

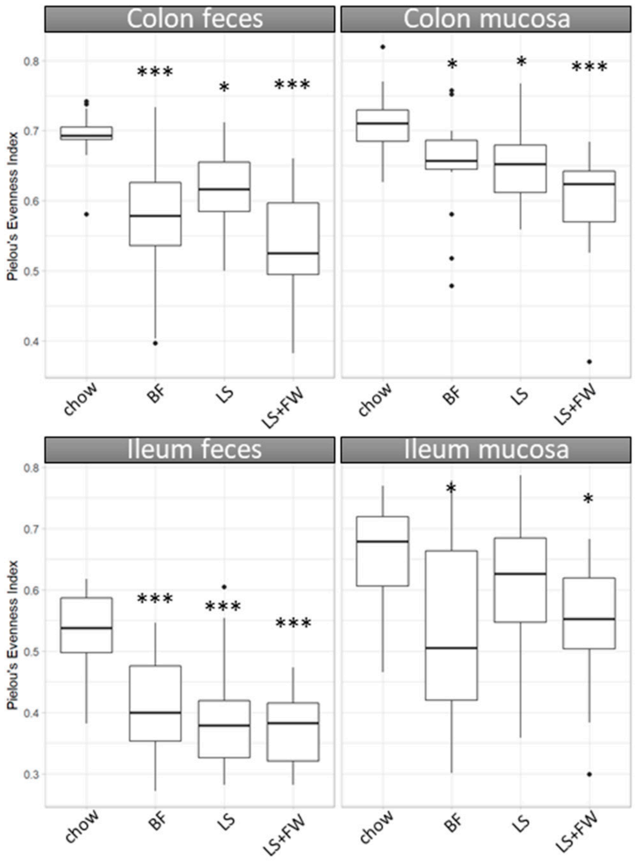

Figure S2. All of the ED diets reduced species evenness in all of the gut compartments. 16S rRNA gene sequencing analysis was performed on the luminal and mucosal contents of both the ileum and colon, and was used to calculate the Pielou evenness index. Data are presented as mean ± SD, * $p \leq 0.05$ or ** $p \leq 0.01$ or *** $p \leq 0.001$ compared to chow.
